# Analysis of the expression characteristics and clinical value of immune function indicators in patients with human immunodeficiency virus infection

**DOI:** 10.12669/pjms.40.10.9895

**Published:** 2024-11

**Authors:** Nannan Wang, Ting Zeng

**Affiliations:** 1 Nannan Wang Department of Laboratory Medicine, Taihe County People’s Hospital, Taihe, Anhui Province, 236600, P.R. China; 2 Ting Zeng, Department of First Class Ward, The First Affiliated Hospital of Jinan University, Guangzhou, Guangdong Province, 510000, P.R. China

**Keywords:** AIDS, Immune function, CD3^+^, CD4^+^, CD8^+^, CD4^+^/CD8^+^, B cells, NK cells

## Abstract

**Objective:**

To explore the expression characteristics and clinical value of immune function indicators in patients with human immunodeficiency virus (HIV) infection.

**Methods:**

In this retrospective study, clinical data of 196 patients with HIV infection (observation group) and 196 health examinees (control group) admitted to our hospital from November 2021 to March 2023 were retrospectively analyzed. Patients in the observation group were further classified based on the staging as acute phase (n=53), asymptomatic phase (n=65), and acquired immunodeficiency (AIDS) phase (n=78). Levels of immune function indicators in the observation group and the control group were compared, and the correlation between immune function index levels and disease staging was analyzed.

**Results:**

Levels of CD3^+^, CD4^+^, CD4^+^/CD8^+^, B cells, and NK cells were lower in the observation group (P<0.05), were significantly lower in asymptomatic patients compared with patients in the acute phase (P<0.05), and were significantly lower in patients with AIDS than those in asymptomatic patients (P<0.05). The results for CD8^+^ were in contrast to the above(P<0.05). Spearman analysis confirmed that levels of CD3^+^, CD4^+^, CD4^+^/CD8^+^, B cells and NK cells significantly negatively correlated, and levels of CD8+ significantly positively correlated with the stages of HIV (P<0.05).

**Conclusions:**

The immune function indicators of patients with HIV infection are markedly abnormal, which is mainly manifested by the decreased levels of CD3^+^, CD4^+^, CD4^+^/CD8^+^, B cells and NK cells, and the increased levels of CD8^+^. The profile of the immune function indicators correlates with the progressive severity of the disease.

## INTRODUCTION

Human immunodeficiency virus (HIV) infection is one of the main causes of morbidity and mortality worldwide.^1^,^2^ HIV is transmitted via infected bodily fluids by sexual contact across mucosal surfaces, by maternal-infant exposure, blood transfusion etc., with the infection rates increasing in recent years.^2^,^3^ Currently, the estimated number of HIV-infected patients worldwide is reaching 38 million, while the number of patients with HIV infection in China is about 1.15 million.^4^,^5^ If untreated, HIV progresses to acquired immunodeficiency (AIDS) within approximately 8-10 years.^6^ Since first being described in early eighties, HIV/AIDS has become a serious public health issue that is associated with a significant socioeconomic burden.^4^,^5^,^7^

After the initial infection, HIV invades host cells via CD4 receptor, causes varying degrees of damage to the immune function that are manifested by abnormally high or low levels of T lymphocyte-related indicators.^8^ If untreated, the infection leads to immune-deficient state which, subsequently, increases the risk of cancer and infection in the immunocompromised patient.^9^ Studies show that levels of CD4^+^, CD8^+^ and other T lymphocyte indicators can accurately reflect the immune function of the body, and can be used to assess the state of illness, disease progression and treatment effectiveness in patients with HIV/AIDS.^8^-^10^ However, there are few stuidies on the association between immune function indicators and stages of HIV infection. This study aimed to explore the expression characteristics and clinical value of multiple immune function indicators (CD3^+^, CD4^+^, CD8^+^, CD4^+^/CD8^+^, B cells, and NK cells) in patients with HIV infection to provide more evidence for clinical management and treatment of HIV.

## METHODS

In this retrospective study, clinical data of patients with HIV infection admitted to our hospital from November 2021 to March 2023 were retrospectively reviewed as the observation group. Meanwhile, clinical data of healthy individuals undergoing physical examinations during the same period were selected as the control group.

### Inclusion & Exclusion Criteria:

Patients in the observation group were further classified based on the staging of HIV infection as acute phase, asymptomatic phase, and AIDS phase.

- Patients aged >18 years old,
- diagnosed with HIV^11^,
- with complete clinical data were included.
- Patients were excluded if;
- with speech communication disorders, mental system disorders, and cognitive impairments;
- with organic lesions in important organs of the body;
- with malignant tumors;
- were breastfeeding and pregnant women;
- with concurrent urinary tract infections;
- received corticosteroids, modulators, interferons, and immunosuppressants in the six months prior to the study;
- with endocrine system disorders.

### Ethical Approval:

The ethical approval was taken from the ethics committee of Taihe County People’s Hospital with No.2023-08, dated October 21^st^ 2023.

Observation indicators were as follows: (1) Basic patient information, including gender, age, BMI, education level, and marital status; (2) Immune function indicators: levels of CD3^+^, CD4^+^, CD8^+^, CD4^+^/CD8^+^, B cells, and NK cells. All patients had 2 ml of peripheral venous blood collected in the early morning on an empty stomach using EDTA anticoagulated negative pressure vacuum blood collection tubes. Serum levels of CD3^+^, CD4^+^, CD8^+^, CD4^+^/CD8^+^, B cells, and NK cells were measured using BD FACSCanto flow cytometry (BD Biosciences, USA) and matched reagents from the same company.

### Statistical Analysis:

SPSS version 26.0 (IBM Corp, Armonk, NY, USA) was used for the analysis. Continuous variables were showed as mean and standard deviation (SD) using student’s t-test for comparison between the two groups. One-way analysis of variance (ANOVA) was used to evaluate the statistical significance of continuous variable differences among patients with different stages of HIV infection, and least significant difference (LSD) method for pairwise post hoc comparison was employed. Count data was showed as number of cases and analyzed using Chi-square test. The correlation between levels of immune function indicators and different disease stages of HIV infection was assessed using Spearman analysis. *P* <0.05 was considered statistically significant. All reported *p*-values were bilateral.

## RESULTS

A total of 196 patients with HIV infection and 196 health examinees were included in this study, and patients in the observation group were further classified as acute phase (n=53), asymptomatic phase (n=65) and AIDS phase (n=78). There was no significant difference in the baseline data between the two groups (*P*>0.05), Table-I. Levels of CD3^+^, CD4^+^, CD4^+^/CD8^+^, B cells, and NK cells in the observation group were lower, while level of CD8^+^ was higher than that of the control group (*P*<0.05), Table-II.

**Table-I:** Comparison of baseline characteristics between two groups.

Group	Gender (male/female)	Age (year)	BMI (kg/m²)	Education level		Marital status	

				Below High School	High school and above	Married	Unmarried
Observation group (n=196)	107/89	51.14±10.31	23.86±3.34	114 (58.16)	82 (41.84)	85 (43.37)	111 (56.63)
Control group (n=196)	120/76	52.53±10.02	24.29±322	104 (53.06)	92 (46.94)	75 (38.27)	121 (61.73)
*χ*^2^/*t*	1.769	-1.347	-1.308	1.033		1.056	
*P*	0.184	0.179	0.192	0.309		0.304	

**Table-II:** Comparison of immune function indicators between the two groups.

Group	CD3^+^ (cells/ul)	CD4^+^ (cells/ul)	CD8^+^ (cells/ul)	CD4^+^/CD8^+^	B cell (cells/ul)	NK cell (cells/ul)
Observation group (n=196)	589±215	528±197	836±163	0.67±0.32	159±59	309±104
Control group (n=196)	935±239	899±178	617±70	1.47±0.32	276±65	495±92
*t*	23.234	35.263	30.612	44.718	24.946	30.572
*P*	<0.001	<0.001	<0.001	<0.001	<0.001	<0.001

Levels of CD3^+^, CD4^+^, CD4^+^/CD8^+^, B cells, and NK cells in asymptomatic patients were significantly lower compared to patients in the acute phase. In contrast, CD8+ levels were significantly higher in asymptomatic patients compared to the acute phase patients (*P*<0.05). Levels of CD3^+^, CD4^+^, CD4^+^/CD8^+^, B cells and NK cells in patients with AIDS were significantly lower than those in asymptomatic patients, while the level of CD8^+^ was significantly higher (*P*<0.05), Table-III. Spearman analysis showed that levels of CD3^+^, CD4^+^, CD4^+^/CD8^+^, B cells and NK cells negatively correlated with the stages of HIV infection (*P*<0.05). In contrast, level of CD8^+^ positively correlated with the stage of the disease (*P*<0.05), Table-IV.

**Table-III:** Comparison of immune function indicators among patients of different stages in the observation group.

Stages of HIV infection	n	CD3^+^ (cells/ul)	CD4^+^ (cells/ul)	CD8^+^ (cells/ul)	CD4^+^/CD8^+^	B cell (cells/ul)	NK cell (cells/ul)
Acute phase	53	818±198	781±159	716±78	1.10±0.22	217±61	428±79
Asymptomatic	65	592±137^a^	501±102^a^	814±139^a^	0.64±0.17^a^	163±35^a^	320±65^a^
AIDS	78	433±122^ab^	378±68^ab^	936±164^ab^	0.41±0.11^ab^	117±32^ab^	218±43^ab^
*F*		102.992	214.669	41.566	267.533	87.245	182.559
*P*		<0.001	<0.001	<0.001	<0.001	<0.001	<0.001

***Note:*** Compared with the acute phase,

a P<0.05; Compared with asymptomatic phase, ^b^P<0.05

**Table-IV:** Analysis of the correlation between immune function indicators and disease staging.

Index		CD3^+^	CD4^+^	CD8^+^	CD4^+^/CD8^+^	B cell	NK cell
Stages of HIV infection	*r*	-0.736	-0.818	-0.553	-0.846	-0.677	-0.814
	*P*	<0.001	<0.001	<0.001	<0.001	<0.001	<0.001

## DISCUSSION

The results of this study indicate that HIV infection is associated with significantly lower levels of CD3^+^, CD4^+^, CD4^+^/CD8^+^, B cells, and NK cells in patients with HIV infection. In contrast, the levels of CD8^+^ are higher in patients with HIV compared to the general population. There is a general consensus that HIV manifests in a certain extent of abnormal immune function that can be evaluated by measuring T lymphocyte subsets and other indicators. Jia et al. investigated the expression characteristics of T lymphocyte subsets in patients with HIV infection, and showed that the infection is associated with abnormal levels of CD4^+^, CD4^+^/CD8^+^, and CD8+, which is consistent with our results.^12^

Previous studies also showed that T lymphocyte levels, specifically, the levels of CD4+ and CD8+ cells, may be used as an indication of the severity of the disease.^12^–^14^ A study by Tibúrcio et al.^15^ that focused on the expression characteristics of CD4^+^ cells in patients with HIV confirmed that it was significantly lower than that in healthy population. The main reason for this abnormal decrease in CD4^+^ cell levels is the continuous accumulation of viral cDNA that has not undergone integration in the cytoplasm, which can cause varying degrees of toxic effects on CD4^+^ cells and ultimately induce cell apoptosis. Moreover, HIV replication and transcription processes can affect protein expression and synthesis of CD4^+^ cells. In addition, studies have shown that HIV-infected CD4^+^ lymphocytes may fuse with uninfected ones to produce multinucleated giant cells, thereby reducing the number of CD4^+^ cells.^15^,^16^ HIV can also infect or inhibit CD4^+^ cells, thymic stromal cells and precursor cells, ultimately inhibiting thymic cell proliferation and growth, and inhibiting CD4^+^ cell secretion and formation.^16^-^18^

A study report by Perdomo-Celis F et al. showed that the level of CD8^+^ in patients with AIDS was abnormally high, which was consistent with the results of this study. Their research also points out that the sustained immune activation and exhaustion caused by the increased burden of antigens and inflammation during HIV infection can induce phenotypic and functional changes, hinder antiviral response, and affect the effectiveness of disease treatment.^19^ Tsukamoto et al. showed that abnormal T lymphocyte function and number play an important role in the process of HIV infection. While CD4^+^ cells play a role in assisting humoral and cellular immunity, CD8^+^ cells can exert inhibitory and killing effects to maintain and induce immune tolerance.^20^

Therefore, number of CD8^+^ T lymphocytes is an important indicator of HIV infection and progression of the disease.^20^,^21^ Lobos et al. demonstrated that abnormal levels of CD4^+^ and CD8^+^ can cause a decrease in cell killing function and dissolution ability, leading to a dominant negative regulation of immune response. This provides favorable conditions for the replication of HIV virus, ultimately leading to the release of cytokines and inflammatory mediators into the bloodstream. Inflammatory response may limit proliferation and differentiation of T cells, inhibit the expression of stimulating molecules, thus affecting initial T cell maturation, upregulating CD8+ expression, and reducing CD4+expression.^22^

Hoang et al.^23^ also showed that the level of CD4^+^ cells in patients with HIV was significantly reduced, while the level of CD8^+^ cells was abnormally increased. The above is consistent with our results. The study also pointed out that CD4^+^ helper T cells can directly participate in the immune response. In case of HIV infection and AIDS, the maturation of CD4^+^ cells is inhibited, leading to the reduced expression levels. In contrast, CD8^+^ cells are T lymphocytes with immunosuppressive function, and therefore, the levels of CD8^+^ cells increase significantly in patients with AIDS.^21^-^23^ This study also investigated the levels of immune function indicators in HIV patients at different stages of the disease. Our results showed a direct correlation between the decreasing levels of CD3^+^, CD4^+^, CD4^+^/CD8^+^, B cells and NK cells and the stage of the disease.

In contrast, levels of CD8^+^ showed an increasing trend, and Spearman analysis confirmed a significant correlation. Our results confirm that all patients with AIDS have impaired immune function, and this immune function defect becomes more significant with the progression of the HIV infection, which is consistent with observations of Li et al.^24^ This study further confirms that the disease progression in AIDS patients may be assessed in clinical practice by measuring the level of immune function-related indicators.

### Limitations:

Firstly, this is a single center retrospective study. The sample size may be prone to selection bias. Secondly, neither group was randomly assigned, and baseline information may be imbalanced and biased, which is also one of the shortcomings of our retrospective study. Higher quality research is needed to verify our observations.

## CONCLUSION

HIV infection is associated with abnormal immune function, which is mainly manifested by the decreased levels of CD3^+^, CD4^+^, CD4^+^/CD8^+^, B cells and NK cells, and the increased levels of CD8^+^. Measuring levels of these indexes may be implemented in the clinical practice to determine the stage of the disease staging, thus providing a reference basis for diagnosis and treatment evaluation.

### Author`s Contribution:

**NW:** Conceived and designed the study.

**NW and TZ:** Collected the data, performed the analysis and Review

**NW:** Was involved in the writing of the manuscript and is responsible for the integrity of the study.

All authors have read and approved the final manuscript.
